# A Novel Role for Brain and Acute Leukemia Cytoplasmic (BAALC) in Human Breast Cancer Metastasis

**DOI:** 10.3389/fonc.2021.656120

**Published:** 2021-04-22

**Authors:** Madeleine Birgersson, Mengna Chi, Chrissy Miller, Joshua S. Brzozowski, Jeffrey Brown, Lachlan Schofield, Olivia G. Taylor, Elizabeth A. Pearsall, Jasmine Hewitt, Craig Gedye, Lisa F. Lincz, Kathryn A. Skelding

**Affiliations:** ^1^ Cancer Cell Biology Research Group, School of Biomedical Sciences and Pharmacy, College of Health, Medicine and Wellbeing, The University of Newcastle, Callaghan, NSW, Australia; ^2^ School of Biomedical Sciences and Pharmacy, Karolinska Intitutet, Solna, Sweden; ^3^ Hunter Cancer Research Alliance and Cancer Research Program, Hunter Medical Research Institute, New Lambton Heights, NSW, Australia; ^4^ Department of Medical Oncology, Calvary Mater Newcastle Hospital, Waratah, NSW, Australia; ^5^ Hunter Hematology Research Group, Calvary Mater Newcastle Hospital, Waratah, NSW, Australia

**Keywords:** breast cancer, migration, invasion, FAK, BAALC

## Abstract

Brain and Acute Leukemia, Cytoplasmic (BAALC) is a protein that controls leukemia cell proliferation, differentiation, and survival and is overexpressed in several cancer types. The gene is located in the chromosomal region 8q22.3, an area commonly amplified in breast cancer and associated with poor prognosis. However, the expression and potential role of BAALC in breast cancer has not widely been examined. This study investigates BAALC expression in human breast cancers with the aim of determining if it plays a role in the pathogenesis of the disease. BAALC protein expression was examined by immunohistochemistry in breast cancer, and matched lymph node and normal breast tissue samples. The effect of gene expression on overall survival (OS), disease-free and distant metastasis free survival (DMFS) was assessed *in silico* using the Kaplan-Meier Plotter (n=3,935), the TCGA invasive breast carcinoma (n=960) and GOBO (n=821) data sets. Functional effects of BAALC expression on breast cancer proliferation, migration and invasion were determined *in vitro*. We demonstrate herein that BAALC expression is progressively increased in primary and breast cancer metastases when compared to normal breast tissue. Increased *BAALC* mRNA is associated with a reduction in DMFS and disease-free survival, but not OS, in breast cancer patients, even when corrected for tumor grade. We show that overexpression of BAALC in MCF-7 breast cancer cells increases the proliferation, anchorage-independent growth, invasion, and migration capacity of these cells. Conversely, siRNA knockdown of BAALC expression in Hs578T breast cancer cells decreases proliferation, invasion and migration. We identify that this BAALC associated migration and invasion is mediated by focal adhesion kinase (FAK)-dependent signaling and is accompanied by an increase in matrix metalloproteinase (MMP)-9 but not MMP-2 activity *in vitro*. Our data demonstrate a novel function for BAALC in the control of breast cancer metastasis, offering a potential target for the generation of anti-cancer drugs to prevent breast cancer metastasis.

## Introduction

Breast cancer is the most common cancer among women worldwide (1). Despite improvements in overall survival rates, it is estimated that approximately 1/3-1/2 of patients, depending on disease subtype, will develop distant metastases (2). Whilst breast cancer overall has an 89% five-year survival rate (1), this reduces to 21% once metastasis has occurred (3). This highlights that the identification of new targets for the treatment of metastatic breast cancer are required.

Amplification of the long arm of chromosome 8, specifically 8q22.3, is a commonly observed genetic alteration in breast cancer (4, 5) and is associated with poor prognosis (6). Several genes within this region have been identified and shown to be associated with prognosis, including *EDD1*, *AZINI*, and *WDSOFI* (6). Another gene, Brain and Acute Leukemia, Cytoplasmic, *BAALC*, is also located in this region, and is overexpressed in a variety of cancer types. However, its expression and function in breast cancer has not been examined until recently, and remains unexplored in non-triple negative subtypes.

The *BAALC* gene was originally identified in blasts from Acute Myeloid Leukemia (AML) patients with trisomy 8 (7), and is normally expressed in the cytoplasm of muscle and brain cells (7) and in CD34+ hematopoietic progenitor cells, but not in peripheral blood or unselected bone marrow cells from healthy donors (8). BAALC is a protein whose function has not been widely studied, however it is abundantly expressed in a range of cancer types, including glioblastoma (7), AML (9–11), melanoma (12), gastrointestinal stromal tumors (13), and triple negative breast cancer (14) suggesting that it may play a role in tumorigenesis. In support of this, BAALC controls cell proliferation in AML (15, 16) and triple negative breast cancer (14) and when BAALC overexpression is combined with the self-renewal promoting oncogene *HoxA9*, leukemogenesis is induced *in vivo* (17). Furthermore, several studies have shown that high *BAALC* expression is a negative prognostic factor in AML (9–11, 18–27), and is associated with shorter disease-free, relapse-free, and overall survival (OS) (26, 28).

BAALC can act as a scaffolding protein in leukemia cells (16), and can induce cell cycle progression of leukaemia cells by interacting with MEK kinase-1 (MEKK1), which inhibits the interaction between extracellular signal-regulated kinase (ERK) and MAP kinase phosphatase 3 (MKP3), thereby leading to sustained ERK activity. However, whether this plays a role in other cancer cell types remains to be determined.

In light of this, the present study examined the expression of BAALC in breast cancer, and characterized the role of BAALC overexpression in breast cancer proliferation, invasion and migration. We demonstrate herein that BAALC is overexpressed in breast cancer, and that this overexpression predicts for shorter OS and distant metastasis free survival (DMFS) for breast cancer patients. We show that breast cancer cell proliferation, invasion and migration is increased with BAALC overexpression, and decreased with BAALC suppression, suggesting that BAALC may be a driver of breast cancer metastasis. Additionally, we show that BAALC can interact with focal adhesion kinase (FAK), an established promoter of tumor progression and metastasis (29), indicating that this BAALC mediated enhancement of breast cancer cell migration and invasion may be controlled by a FAK-dependent mechanism.

## Materials and Methods

### Tissue Microarray

Tissue microarrays were purchased from SuperBioChip Laboratories (Seoul, South Korea), and were comprised of 40 matched normal breast cores, 70 breast cancer cores, and 10 matched lymph node cores. The tissues were stained for BAALC expression with a rabbit monoclonal antibody (1:50; Novus Bio, Noble Park North, VIC, Australia), using a Ventana Discovery Ultra automated system (Ventana Medical Systems Inc., Tucson, AZ, USA) as previously described (30), with the following modifications. Antigen retrieval was performed for 40 min, and Biocare Medical Background Sniper was applied for 32 mins at 35°C. Sections were scored using an immunohistochemistry score on a continuous scale of 0-300, as previously described (31). Briefly, this involves integrating the intensity and frequency of staining. Staining intensity was scored in four categories: no staining (0), weak staining (1, light brown membrane staining that is visible only under high magnification), intermediate staining (2), and strong staining (3, dark brown staining that is visible under low magnification). The percentage of cells at different staining intensities was determined using HALO software (Indica Labs, Corrales, NM). An H-score was then calculated using:

1×(percentage of cells with weak staining)+2×(percentage of cells with intermediate staining)+3×(percentage of cells with dark staining)

### Bioinformatics Analysis of *BAALC* Expression

Retrospective Kaplan–Meier OS and DMFS analyses of 3,935 human patients with invasive breast cancer were performed using the Kaplan-Meier plotter database (32). Patients were divided into two groups according to *BAALC* gene expression level, and the best performing threshold was used as cutoff for the Kaplan–Meier analyses. Association of outcome was investigated for the total patient cohort with OS or DMFS as end points.

Patients from the TCGA (Firehouse Legacy) data set consisting of 960 patients with invasive breast carcinoma were divided into two groups based on alterations in *BAALC* expression, as queried with cBio Cancer Genomic Portal (http://cbioportal.org), an online web resource used to explore, visualize and analyze multidimensional cancer genomics data (33). mRNA expression z-scores relative to all samples (log RNA Seq V2 RSEM) with a z-score threshold ± 2.0 were used. Association of outcome was investigated for the total patient cohort with disease-free survival as the end point.

Bioinformatic analysis of *BAALC* was assessed using data from the gene expression based outcome for breast cancer online algorithm (GOBO) (34). Association of outcome was investigated for the untreated patient cohort (n=821), with DMFS as end point, with and without multivariate analysis, using estrogen receptor (ER)-status, age, tumor size, and stratified histological grade as covariates, and with no time-dependent censoring. P-value is calculated using the log-rank test.

### Cell Lines and Manipulation of BAALC Expression

MCF-7 (ATCC HTB-22) and Hs578T (ATCC HTB-126) cells were purchased from the American Type Culture Collection (ATCC; Manassas, VA, USA) immediately prior to commencing this study. Cells were maintained in DMEM, supplemented with 15 mM HEPES, 2 mM glutamine, and 10% fetal calf serum (FCS), with additional 0.01mg/ml bovine insulin for Hs578T. All cell culture reagents were purchased from Thermo Fischer Scientific (Mulgrave, VIC, Australia). MCF-7 cells were stably transduced with either empty vector (EV) or full length BAALC (Lv41-BAALC) pseudoviral particles (GeneCopoeia, Rockville, MD, USA), as per manufacturer’s instructions.

Hs578T cells were transiently transfected with siRNA directed against BAALC (or scrambled control sequences) by Lipofectamine 3000 (Thermo Fisher Scientific) as previously described (35). Pools of three to five target-specific 19-25 nucleotide siRNAs designed to knockdown BAALC expression (and control siRNA sequences) were purchased from Santa Cruz Technology (Dallas, Tx, USA). Cells were then lysed at various times post-transfection or used in functional assays to measure effects on proliferation, migration and invasion, as described below.

### Scratch Migration Assay

Scratch wound migration assays were conducted using stably transfected MCF-7 cells. Confluent monolayers of transfected MCF-7 cells in 24-well plates were serum starved for 24 h to induce quiescence, and were then treated for 1 h with either serum free media, vehicle control, 70 nM UO126 (1,4-diamino-2,3-dicyano-1,4-*bis*(2-aminophenylthio)butadiene; Abcam, Melbourne, VIC, Australia), or 1.5 nM PF-562271 (*N*-Methyl-*N*-[3-[[[2-[2-oxo-1,3-dihydroindol-5-yl)amimo]-5-(trifluoromethyl)pyrimidin-4-yl]amino)pyridine-2-yl)methanesulfonamide; Abcam). Following this, a scratch wound migration assay was performed as previously described (30).

### Transwell Migration and Invasion Assays

The migratory properties of transfected MCF-7 and Hs578T cells were investigated using 8 µm pore transwells (Sigma-Aldrich, Castle Hill, NSW, Australia) as previously described (30).

The invasive properties of transfected MCF-7 and Hs578T cells were investigated as above, with the following modification. Prior to the addition of cells, transwell chambers (8 µm pore) were coated with 100 µL 0.6 mg/mL Matrigel (BD Biosciences) solution in serum free media overnight at 37°C, before excess media was removed. Cells (5 x 10^4^ cells/chamber in serum free media) were added to the top chambers, and DMEM supplemented with 10% FCS was added to the lower chambers.

### Cell Titre Blue Assay

A growth assay was performed as previously described (35, 36). Briefly, 1 x 10^4^ transfected MCF-7 and Hs578T cells were seeded per well, and at various times post plating, proliferation was assessed by measuring resazurin reduction using the Cell Titer-Blue Cell Viability Assay (Promega, Alexandria, NSW, Australia), as per manufacturer’s instructions.

### Tumorigenic Assay

The tumorigenicity of transfected MCF-7 cells was measured by plating 2 x 10^3^ cells in growth medium. After 14 days incubation, colonies were stained with 0.05% crystal violet/10% methanol/phosphate buffered saline (PBS) for 30 min and counted. Three independent experiments were performed, with assays being plated in triplicate.

### Soft Agar Assay

Anchorage-independent growth of transfected MCF-7 cells was measured as previously described (30). Briefly, 5 x 10^3^ cells were plated in semisolid culture media (1.2% methylcellulose; Sigma-Aldrich) supplemented with 20% FCS, on a layer of 0.7% agar in growth medium as previously described (30).

### Western Blotting

Cells were lyzed in lysis buffer and homogenized as previously described (37). Cell lysate (20 µg) were separated using 4-12% Bis-Tris-polyacrylamide gel electrophoresis (PAGE, Life Technologies) in 1 x MES buffer (Life Technologies), and then transferred to nitrocellulose membranes for 1 h at 4°C at 300 mA, using a Tris-glycine-methanol transfer buffer (48 mM Tris, 39 mM glycine, 10% methanol, pH 8.3), and western blot performed as described previously (37, 38). The primary antibodies used were as follows: BAALC (7.3_1E10; 1:1,000 overnight at 4°C; Abcam), pY397-focal adhesion kinase (FAK; 1:1,000 overnight at 4°C; Abcam), FAK (EP695Y; 1:2,000 overnight at 4°C; Abcam), pT202/pY204-extraellular signal-regulated kinase (ERK)1 and pT185/Y187-ERK2 (1:5,000 overnight at 4°C; Abcam), ERK1/ERK2 (1:1,000 overnight at 4°C; Abcam) and GAPDH (1:2,000 for 1 h at room temperature, BioVision, Alexandria, NSW, Australia).

### Zymography

Protease activity of transfected MCF-7 cells was assessed using 10% gelatin zymogram gels (Thermo Fisher), as previously described (39).

### Immunoprecipitation

The interaction between BAALC and FAK was determined by immunoprecipitation. MCF-7-EV and MCF-7-BAALC cells (1 x 10^6^) were pelleted and lyzed as described above. Lysates (500 µg) were incubated with BAALC antibody (7.3_1E10; 1:1000; Abcam), a FAK antibody (12G4; 5 µg/mL; Abcam), or an IgG control antibody (1:1000; Santa Cruz Biotechnology, Dallas, TX, USA) and 100 µL of Protein A/G agarose overnight at 4°C, with gentle rotation. After this time, the beads were washed 3 times (5 mins each) in TBST to remove any unbound protein. Beads were then boiled for 5 min in SDS sample buffer, and BAALC and FAK detected by western blotting.

### Data Analysis

All statistical analyzes were conducted using GraphPad Prism software V8.0 (GraphPad, San Diego, CA, USA). All *in vitro* functional comparisons were made using an unpaired two-tailed t-test. Immunohistochemistry data comparisons were made using a one-way analysis of variance (ANOVA), with a Tukey’s multiple comparisons test. Data is presented as the mean ± standard error of the mean (SEM) for the number of replicates (n).

## Results

### BAALC Is Over-Expressed in Breast Cancers and High Expression Predicts Poor Patient Outcome

**Figure 1:**
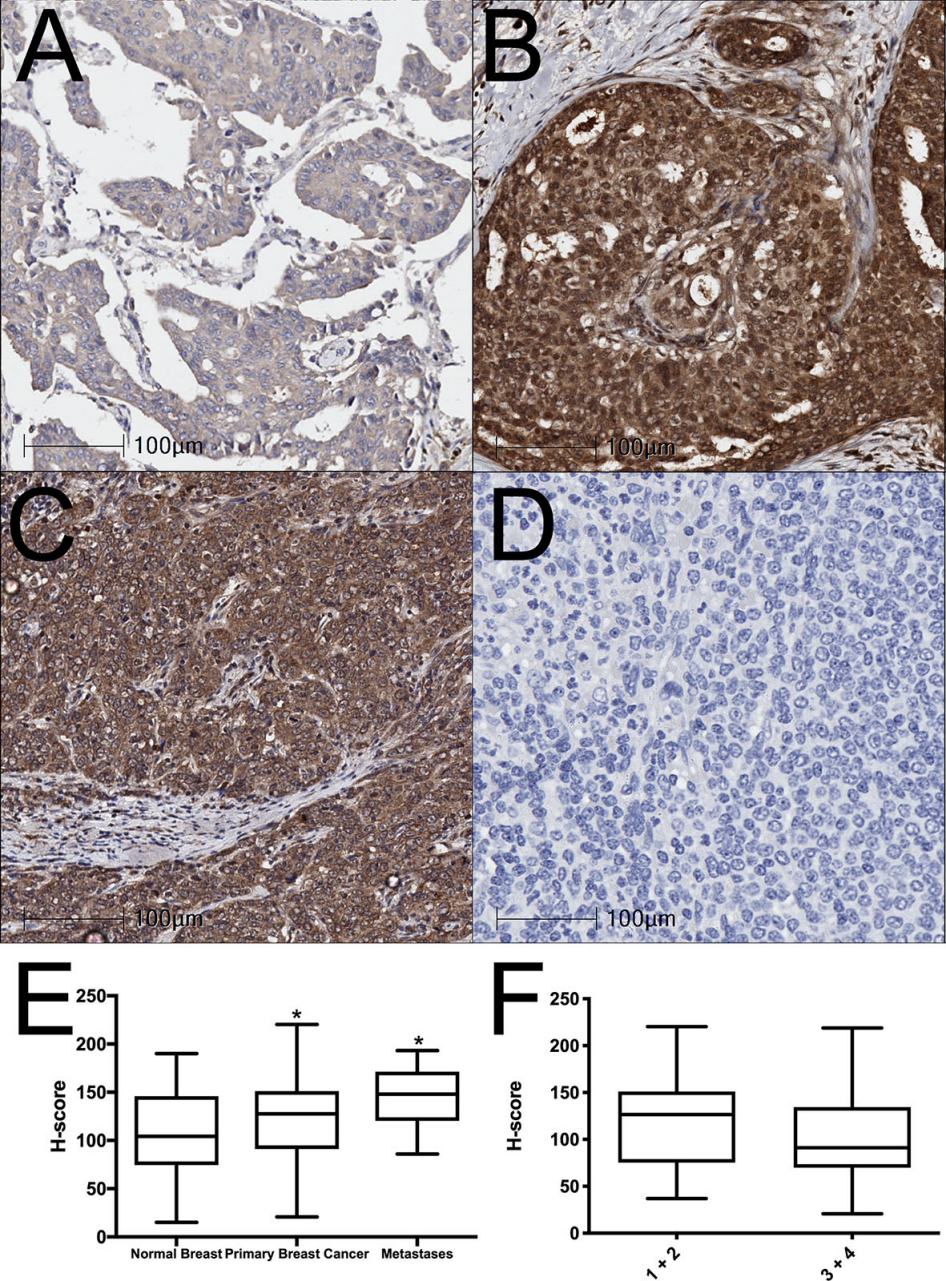
BAALC expression is increased in primary breast cancer and lymph node metastases tissues. **(A)** Normal breast, **(B)** primary breast cancer, and **(C)** lymph node metastases were examined for BAALC expression by immunohistochemistry. **(D)** Negative control. **(E)** Staining was quantified and expressed as an H-score in 70 primary breast cancer, 40 matched normal breast, and 10 lymph node metastases cores, **(F)** H-score comparison between tumors classified as Grade 1 + 2 or Grade 3 + 4 from n=70 primary breast cancer in **(E)** Photomicrographs are representative of each tissue type. * denotes statistical significance p < 0.05 as determined by one-way ANOVA.

BAALC expression was examined in normal human breast, breast cancer, and lymph node metastasis tissue by immunohistochemistry. BAALC expression was scored on a scale of 0-300, as previously described (31). The mean expression of BAALC was significantly higher in breast cancer (**Figures 1B, D**; p = 0.0459) and metastases (**Figures 1C, D**; p = 0.0206) compared to normal breast tissue (**Figures 1A, D**). However, there was no significant difference in BAALC expression between low and high grade tumors (grades 1 + 2 vs 3 + 4; **Figure 1F**, p=0.2031).

**Figure 2:**
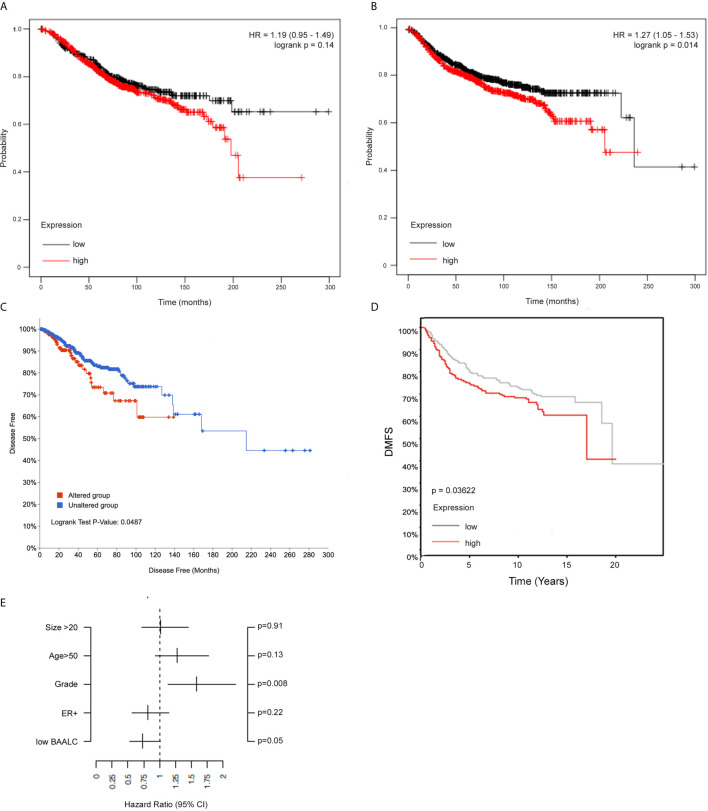
High *BAALC* predicts for worse progression free and distant metastasis free survival in breast cancer patients. Kaplan-Meier curves showing the **(A)** overall survival and **(B)** distant metastasis-free survival in a publicly available 3,935-sample breast cancer data set (32) with elevated (red) or low (black) expression of *BAALC* in breast cancer tumors when assessing all tumor subtypes together. Kaplan-Meier curves showing the **(C)** disease-free survival in a publicly available 960-sample breast cancer data set (33), with high (red) or low (blue) expression of *BAALC*. Kaplan-Meier curves showing the **(D)** distant metastasis free survival in a publicly available 821-sample breast cancer data set (34), with high (red) or low (gray) expression of *BAALC*. **(E)** Multivariate analysis for the Kaplan-Meier analysis shown in **(D)**, using estrogen receptor (ER)-status, tumor size, age, histological grade and low BAALC as covariates, distant metastasis free survival as endpoint with no time-dependent censoring. The hazard ratio and the 95% confidence interval are plotted for each of these covariates. p values were computed by a log-rank test.

We next assessed whether BAALC expression was associated with breast cancer patient outcome by investigating *BAALC* mRNA expression in a publicly available 3,935-sample breast cancer data set, where high *BAALC* expression was not linked to OS (**Figure 2A**, p=0.14), but specifically associated with significantly worse DMFS (**Figure 2B**, p=0.014) (32). This was confirmed in two additional cohorts, a 960-sample invasive breast cancer cohort which showed that high *BAALC* expression was significantly associated with worse disease-free survival (**Figure 2C**; p = 0.0487) (33), and in an 821-sample untreated breast cancer cohort demonstrating that high *BAALC* expression was significantly associated with worse DMFS (**Figure 2D**; p = 0.03622). When corrected for potentially confounding factors of tumor size, grade, ER status, and patient age, BAALC expression was the second most important contributor to DMFS in this cohort, with a p-value bordering on significance (p=0.05; **Figure 2E**), suggesting that BAALC may be a potential prognostic factor that is independent of tumor grade.

### BAALC Overexpression Enhances Proliferation and Anchorage-Independent Growth of MCF-7 Breast Cancer Cells

**Figure 3:**
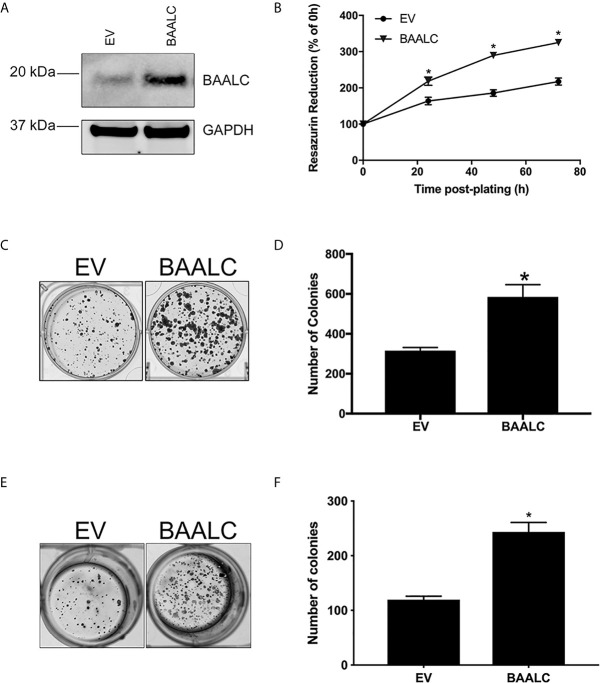
BAALC overexpression increases breast cancer cell proliferation and anchorage-independent growth *in vitro*. MCF-7 cells stably transfected with an empty vector (EV) or BAALC were generated. **(A)** *Top* BAALC expression was confirmed by western blot, *bottom* GAPDH as a loading control. **(B)** Cell viability was measured at 0, 24, 48, and 72 h post-plating *via* Cell Titre Blue Assay. n=3 in triplicate. **(C)** Transduced MCF-7 cells were grown for 14 days. After this time colonies were stained with 0.5% crystal violet/10% methanol/PBS for 30 mins. Photomicrographs are representative of three independent experiments, performed in triplicate. **(D)** Colonies were counted. **(E)** Transduced MCF-7 cells were grown for 15 days in soft agar before the colonies were stained with 0.5% crystal violet/10% methanol/PBS for 30 mins. Photomicrographs are representative of four independent experiments, performed in duplicate. **(F)** After staining, the colonies in each well were counted. * denotes statistical significance p < 0.05, as determined by an unpaired two-tailed t-test.

We therefore next examined whether BAALC overexpression could alter processes involved in the metastasis of breast cancer cells. A full-length form of BAALC was transfected into MCF-7 breast cancer cells, which have low levels of endogenous BAALC expression. Empty vector (EV) control MCF-7 cells expressed minimal levels of endogenous BAALC, whilst cells stably transfected with BAALC showed increased levels of BAALC (**Figure 3A**).

As BAALC regulates leukemia cell proliferation *in vitro* (15, 16), we firstly examined the effects of BAALC overexpression on the proliferative capacity of MCF-7 breast cancer cells. BAALC overexpression significantly increased cell proliferation, when compared to EV cells, as measured by Cell Titre Blue (**Figure 3B**; p < 0.0001) and clonogenic assays (**Figures 3C, D**; p = 0.0128).

As the ability of cancer cells to grow without adhering to extracellular matrix (ECM) is associated with tumorigenicity in animal models (40), we next examined the effect of BAALC overexpression on anchorage-independent growth of MCF-7 cells. BAALC overexpression significantly enhanced the ability of MCF-7 cells to grow in a semi-solid medium, when compared to EV control cells (**Figures 3E, F**; p < 0.0001).

### BAALC Overexpression Promotes Migration and Invasion of Breast Cancer Cells

**Figure 4:**
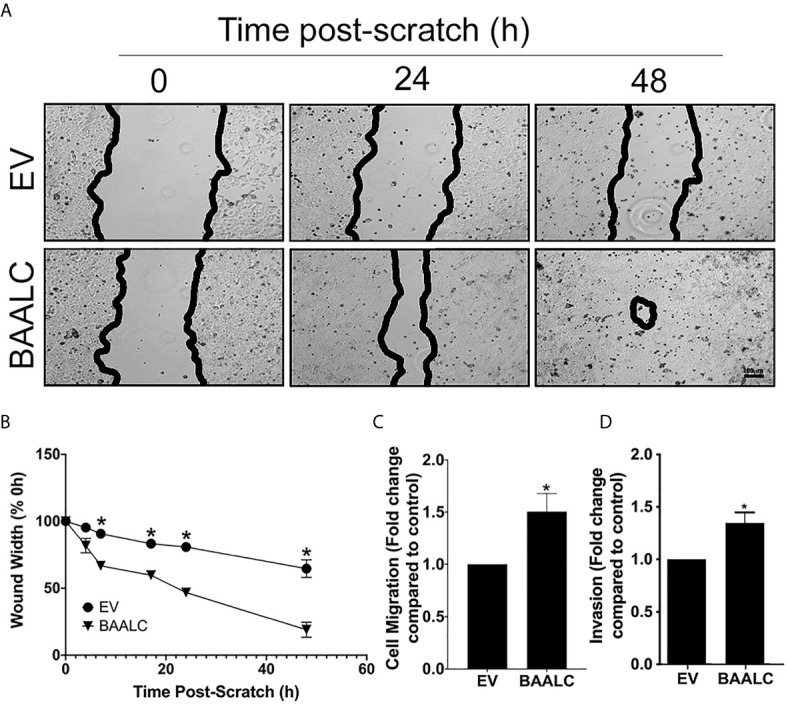
BAALC overexpression increases MCF-7 migration *in vitro*. MCF-7 cells stably transfected with an empty vector (EV) or BAALC were generated. **(A)** Confluent monolayers of MCF-7-EV and BAALC cells were grown to confluence, and a wound was made by scratching with a pipette tip. The wounds were photographed at 4, 7, 17, 24 and 48 h to measure wound closure over time. Photomicrographs are indicative of 6 independent experiments performed in triplicate. **(B)** Wound widths are expressed as % of 0 h wound width. * denotes statistical significance (p < 0.05). **(C)** MCF-7 cells expressing BAALC or EV were placed in the upper chamber of a Transwell and allowed to migrate through the uncoated membrane (8 µm pore) for 4 h. n=3 performed in triplicate. **(D)** MCF-7 cells expressing BAALC or EV were examined for ability to invade through Matrigel plugs. n=3 performed in triplicate. * denotes statistical significance p < 0.05.

MCF-7 cells overexpressing BAALC migrated significantly more rapidly than EV cells in a wound healing assay (**Figures 4A, B**; p = 0.0004). To confirm that these effects were not due to enhanced proliferation, a short-term (4 h) transwell migration assay was also performed, and BAALC overexpression once again significantly increased MCF-7 migration (**Figure 4C**; p = 0.0443).

To determine whether BAALC overexpression also enhanced the invasive capacity of MCF-7 cells, an invasion assay through Matrigel plugs was performed. Significantly larger numbers of MCF-7 cells overexpressing BAALC invaded through Matrigel plugs when compared to EV control cells (**Figure 4D**; p = 0.025).

### Decreased BAALC Expression Inhibits Proliferation, Migration and Invasion of Hs578T Breast Cancer Cells

**Figure 5:**
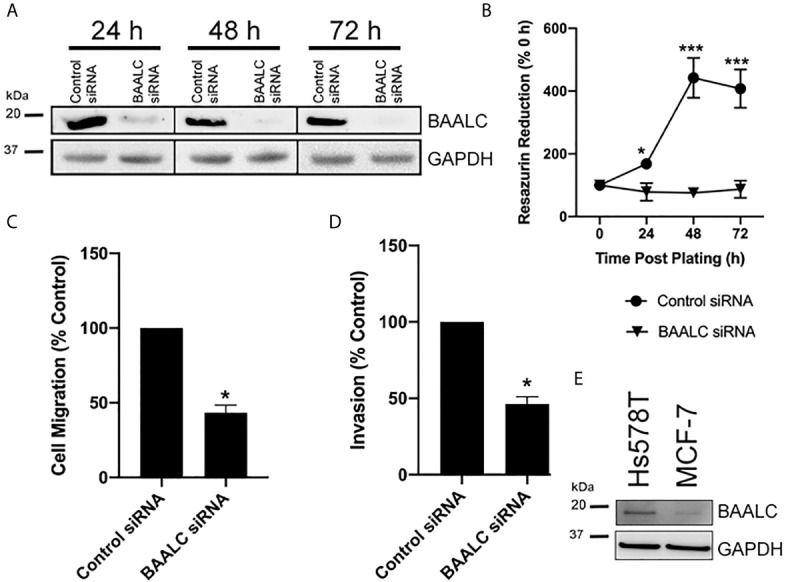
Knocking down BAALC expression decreases Hs578T proliferation, migration and invasion *in vitro*. Hs578T cells were transfected with a pool of siRNA directed against BAALC, or a controlled scrambled sequence. **(A)** *Top* BAALC expression at various times post-transfection was examined by western blot, *bottom* GAPDH as a loading control. **(B)** Cells were plated immediately following transfection and cell viability was measured at 0, 24, 48, and 72 h post-plating *via* Cell Titre Blue Assay. n=3 in triplicate. **(C)** Forty-eight hours post-transfection, cells were placed in the upper chamber of a Transwell and allowed to migrate through the uncoated membrane (8 µm pore) for 4 h. n=3 performed in triplicate. **(D)** Forty-eight hours post-transfections, cells were examined for ability to invade through Matrigel plugs. **(E)** Endogenous levels of BAALC protein in Hs578T and MCF7 cells. n=3 performed in triplicate. * denotes statistical significance p < 0.05. *** denotes statistical significance p < 0.001.

As overexpression of BAALC enhanced proliferation, migration and invasion, we next examined the effects of decreasing BAALC expression. Hs578T cells were chosen for these experiments, as they have naturally high expression of BAALC compared to MCF-7 (**Figure 5E**). Whilst control siRNA transfected Hs578T cells expressed high levels of endogenous BAALC, cells transfected with siRNA directed against BAALC showed decreased levels of BAALC at 24, 48 and 72 h post-transfection (**Figure 5A**). Reducing BAALC expression significantly reduced cell proliferation from 24 h post-transfection (24 h, p = 0.0157; 48 h, p < 0.0001; 72 h, p = 0.0004), when compared to control cells, as measured by Cell Titre Blue (**Figure 5B**). Seventy-two hours post knock-down cells began to undergo apoptosis (data not shown).

To determine whether knocking down BAALC expression also suppressed the invasive and migratory capacity of Hs578T cells, short-term (4 h) transwell migration and invasion through Matrigel plug assays were performed. Decreasing BAALC expression significantly reduced Hs578T migration compared to control transfected cells (**Figure 5C**; p = 0.0004). Reducing BAALC expression significantly decreased the number of Hs578T cells invading through Matrigel plugs when compared to control transfected cells (**Figure 5D**; p = 0.0004).

### BAALC Can Interact With FAK, and BAALC Overexpression Is Associated With Expression of Active MMP-9

**Figure 6:**
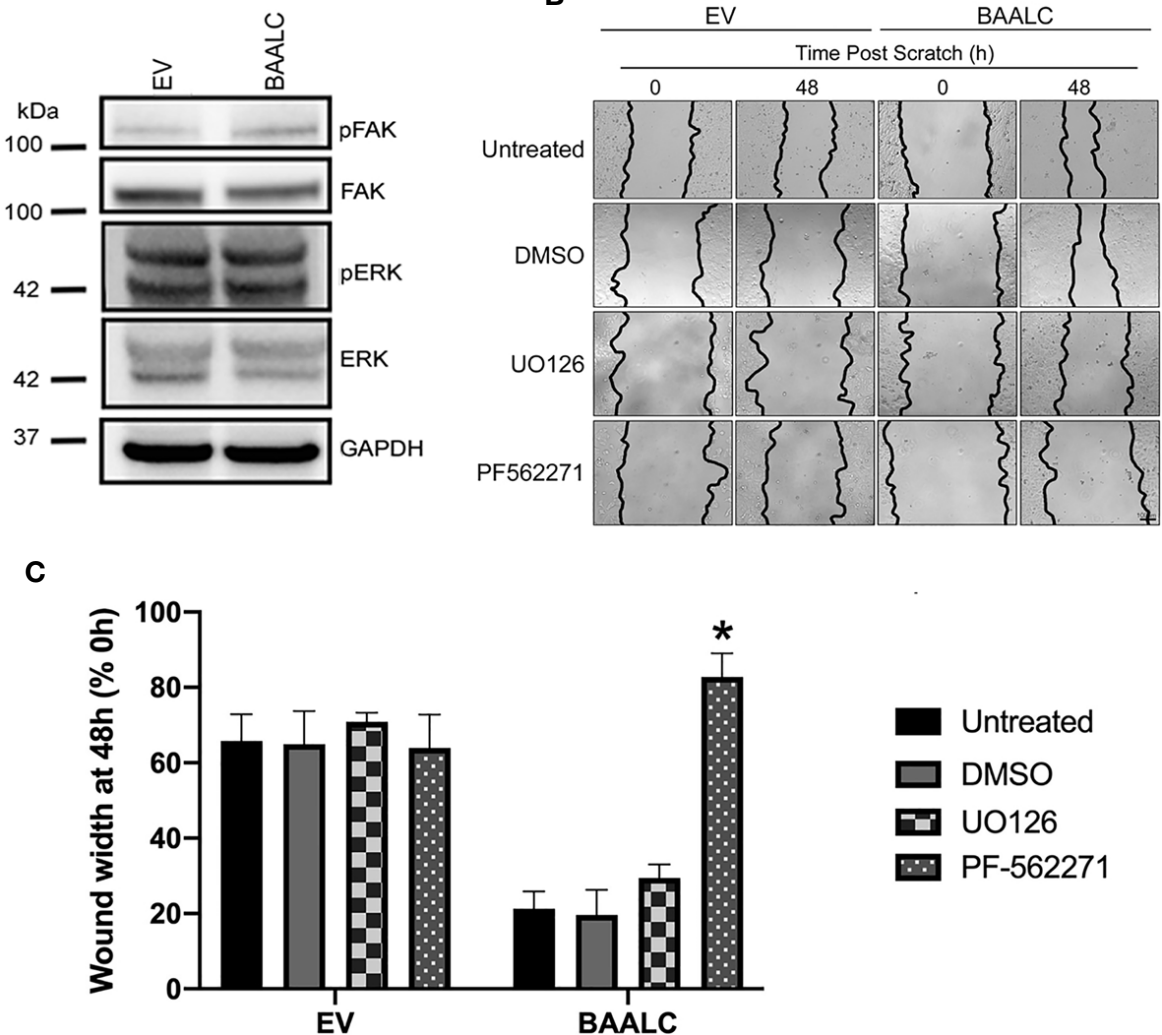
FAK, but not ERK, inhibition decreases BAALC-mediated MCF-7 migration *in vitro*. MCF-7 cells stably transfected with an empty vector (EV) or BAALC were generated. **(A)** The expression and phosphorylation of FAK and ERK following BAALC overexpression were measured by western blot in MCF-7-EV and BAALC cells. Blots are representative of three independent experiments. **(B)** Confluent monolayers of MCF-7-EV and BAALC cells were grown to confluence, treated for 1 h with either serum-free media (untreated), vehicle, UO126 or PF-562271, and a wound was made by scratching with a pipette tip. The wounds were photographed at 0 and 48 h to measure wound closure. Photomicrographs are indicative of 4 independent experiments performed in triplicate. **(C)** Wound widths are expressed as % of 0 h wound width. * denotes statistical significance (p < 0.05).

**Figure 7:**
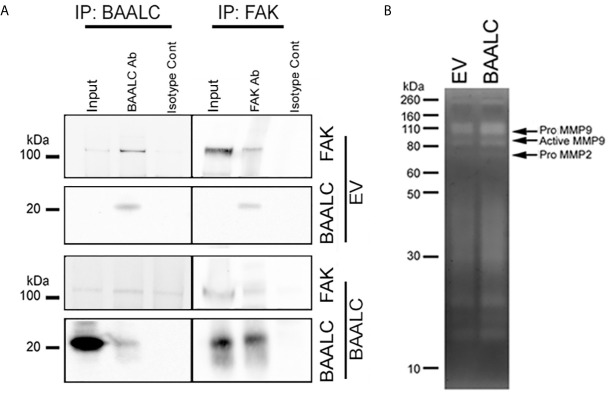
BAALC can interact with FAK and leads to increased active MMP-9 *in vitro*. MCF-7 cells stably expressing an empty vector (EV) or BAALC were generated. **(A)** MCF-7 cells were lyzed, and BAALC was immunoprecipitated from cell lysates with an anti-BAALC antibody, or an IgG isotype control. Co-immunoprecipitated FAK was identified by western blot. Alternatively, endogenous FAK was immunoprecipitated with an anti-FAK antibody, and co-immunoprecipitated BAALC identified by western blot. Blots are representative of three independent experiments. **(B)** Conditioned media from MCF-7-EV and BAALC cells was electrophoresed on 10% gelatin zymography gels, and gels were stained before being visualized for MMP activity. Gels are representative of three independent experiments.

As several pathways are known to mediate breast cancer metastasis, including FAK and ERK (41, 42), and BAALC potentiates the ERK pathway in AML cells (16), we examined expression and phosphorylation of FAK and ERK in EV and BAALC overexpressing MCF-7 cells. BAALC overexpressing cells exhibited increased phosphorylation of FAK, but not ERK, compared to EV control cells (**Figure 6A**). In addition, MCF-7-BAALC cells treated with a FAK inhibitor (PF-562271), but not an ERK inhibitor (UO126), exhibited decreased wound healing when compared to vehicle or untreated control cells (**Figures 6B, C**), with migration levels returning to those of MCF-7-EV cells. Finally, reverse co-immunoprecipitation revealed a direct association between FAK and BAALC in these breast cancer cells (**Figure 7A**), suggesting that BAALC overexpression may be enhancing breast cancer cell migration *via* a FAK-mediated pathway.

Phosphorylation of FAK has been associated with expression of the matrix metalloproteinase (MMP)-9 (42–44). MMP-9 and MMP-2 are implicated in the metastasis of breast cancers *via* degradation of the ECM and promotion of tumor vascularisation (45, 46). Gelatin zymography showed that MCF-7 cells secrete both pro- and active MMP-9 activity (**Figure 7B**), which were enhanced in BAALC overexpressing cells. By contrast, MCF-7 cells expressed pro-MMP-2 but not active MMP-2, irrespective of BAALC expression.

Taken together, these results suggest that BAALC overexpression in breast cancer cells leads to phosphorylation of FAK and secretion of MMP-9, resulting in increased capacity for migration and invasion and ultimately higher risk of metastasis.

## Discussion

BAALC is abundantly expressed in several cancer types, including acute leukemias (9, 10), glioblastoma (7), gastrointestinal stromal tumors (13) and melanoma (12). It has been most widely studied in leukemia, where it has been established as an independent prognostic factor (9, 10, 24, 26, 47), and as a regulator of AML cell proliferation and survival (15, 16). However, its prognostic value and functions in other cancer types has remain largely unexplored. Our data presented herein show that BAALC is overexpressed in primary breast cancer and lymph node metastases compared to normal breast tissue (**Figure 1**), suggesting that it may play a role in breast cancer progression. Indeed, increased *BAALC* mRNA was associated with significantly worse PFS and DMFS in breast cancer patients (**Figure 2**), indicating that high *BAALC* mRNA is a potential novel biomarker for breast cancer patient outcome. However, the precise cellular functions controlled by BAALC in breast cancer cells remains largely unexplored.

Whilst BAALC overexpression alone has little effect on the proliferation of normal cells (17), a role for BAALC in controlling the proliferation of AML cells (15, 16) has been demonstrated. We show herein that BAALC overexpression also increases breast cancer cell proliferation (**Figure 3**), indicating that this BAALC-mediated enhancement of proliferation is not restricted to hematopoietic cells. Conversely, decreasing BAALC expression suppresses proliferation (**Figure 5B**) and induces apoptosis (data not shown). Mechanistically, Morita et al. (16) have demonstrated that this proliferative effect in AML cells is mediated by BAALC potentiating the oncogenic ERK pathway. By contrast, following BAALC overexpression in MCF-7 breast cancer cells, we did not observe an increase in ERK activation (**Figure 6**), indicating that the BAALC overexpression induced increase in proliferation is not mediated *via* the oncogenic ERK pathway in MCF-7 cells.

As we identified that increased *BAALC* mRNA expression was associated with significantly worse DMFS (**Figure 2**), we examined migration and invasion following BAALC overexpression in MCF-7 breast cancer cells. Herein, we show that BAALC overexpression increases breast cancer cell invasion, migration (**Figure 4**) and anchorage-independent growth (**Figure 3**). By contrast, decreasing BAALC expression in Hs578T cells decreased breast cancer cell migration and invasion (**Figures 5C, D**). Li et al. have recently identified a role for BAALC overexpression in controlling triple negative breast cancer cell proliferation and invasion (14), and our results confirm this finding in an additional triple negative breast cancer cell line as well as extend this finding to luminal A breast cancer cells. Taken together with our data demonstrating that BAALC is overexpressed in metastases (**Figure 1**), this indicates that BAALC overexpression may enhance breast cancer metastasis.

As BAALC acts as a scaffolding protein in leukemia cells (16), we identified that BAALC binds to FAK (**Figure 7**), which may explain the pro-migratory and invasive properties of BAALC overexpression in breast cancer cells. FAK is an established promoter of tumor progression and metastasis (29). Autophosphorylation of FAK at Y397 promotes MCF-7 breast cancer cell motility and invasion (48), and Y397 phosphorylation and FAK kinase activity are required for the generation of an invasive cell phenotype (49). We show herein that BAALC overexpressing cells exhibit significantly higher levels of p-Y397 FAK (**Figure 6A**), and that inhibiting FAK, but not ERK, can decrease the enhanced BAALC-mediated breast cancer cell migration (**Figures 6B, C**). However, as FAK inhibition did not show a significant difference in wound width compared to the untreated EV control cells, endogenous migration may be FAK-independent, or BAALC may be required for the FAK-dependent effect in breast cancer cell invasion to be observed.

MMPs are a family of enzymes that play an essential role in cancer, particularly for invasion through extracellular matrices (50). MMP-9 and FAK are associated with MDA-MB-231 breast cancer cell growth factor mediated motility and invasion (42, 43). BAALC overexpression led to an increase in the expression of active MMP-9, but not active MMP-2, compared to EV expressing MCF-7 breast cancer cells (**Figure 6A**). Taken together, our data indicate that BAALC directly interacts with FAK, and that BAALC overexpression led to an increase in the expression of active MMP-9 suggesting that BAALC may enhance breast cancer cell migration and invasion through a FAK and MMP-9-mediated mechanism.

In conclusion, we have identified a new mechanism for enhancing breast cancer metastasis, specifically BAALC overexpression. BAALC overexpression enhances breast cancer cell proliferation, invasion and migration *in vitro*. This study raises the possibility that BAALC may be a novel prognostic marker for breast cancer, and also a target for controlling breast cancer cell metastasis.

## Data Availability Statement

The original contributions presented in the study are included in the article. Further inquiries can be directed to the corresponding author.

## Ethics Statement

The studies involving human participants were reviewed and approved by University of Newcastle Human Research Ethics Committee.

## Author Contributions

MB, MC, JB, CM, OT and JH performed and analyzed the invasion, migration, and proliferation assays. MB, MC, JB and CM performed the western blots. LS, JB and LL performed the zymography. CG and CM performed the survival analysis. JB, CM and EP performed the immunoprecipitation experiments. KS designed and directed the study. All authors contributed to the article and approved the submitted version.

## Funding

This work was supported by research funds from the Cure Cancer Australia Foundation, Tour de Cure, the Hunter Medical Research Institute, the Hunter Translational Cancer Research Centre, the Hunter Cancer Research Alliance, and the University of Newcastle.

## Conflict of Interest

The authors declare that the research was conducted in the absence of any commercial or financial relationships that could be construed as a potential conflict of interest.
